# The Practice of Dentistry in Germany

**Published:** 1885-08

**Authors:** Emil Amend

**Affiliations:** Student in Dental Department, University of Maryland.


### Editorial, Etc.

The Practice of Dentistry in Germany.—*Separate copy from the Cologne paper of the 5th of Nov. 1884.*—The instalment of the liberty of trade has led to the largest consequences in regard to all the medical science. In all German countries in former times only they had the right to practice who had passed examination before the board of government. Now after the fixation of the German order for trade of the year 1869 is the practice of medical science not more bound to a proof of the capability, but everybody is allowed to practice the "physician's" trade, only the sign of the right as "physician" (pract. physician, surgeon, dentist, Zahnarst and so forth) is dependent from the possession of an approbation. Besides, of that possession of right, got in foreign countries, every mark as physician, etc., is allowed, if here is only added to it, "approbated in foreign countries."

This license of the physician's helping hand and the right to bear a title, got in foreign countries, has not been without the doubtfullest consequences in Germany. You may particularly see, that, in the branch of dentistry we have entered here, in situations that cannot remain so for the future. The number of those persons who have not enough. and no prefiguration at all, is now very large. They are using and practising mostly under the title as "Dentist, Zahnartist, Zahnoperator, Zahntechnickereh." Thus you will find at Berlin more than 160 of such "physicians" and yet not 60 dentists approbated in Germany. Those teeth-operators, etc., who use the liberal legislation for the disadvantage of suffering mankind, have been either helping hands at dentists' or barbers' offices, or they have got their education in one of these private institutions that make a good deal of money by instructing young men in Dentistry in 4—5 weeks, so far that they may practice for themselves.

Now; besides those persons who practice Dentistry like a handicraft, there are still a number of "physicians" viz: the dentists "approbated in foreign countries," who take this title to avoid German examination for dentists. The most of these dentists have got their "approbation" from the U. S., of North America. It is well known, for a long time, to all German dentists, (in the circle of the profession) what mischief is done by the American dental colleges with the conferring of diplomas, particularly to Germans, by taking every foreignor into the college, without any regard to his education and capability if he only pays the high fees; then they will give to him, or everyone of them, the diploma as "doctor of dental surgery" after 4—5 months being passed. That these "studies," as well as the examination made by help of an interpreter, does not deserve that name, when we know that the most American dentists in our fatherland do not understand the English language; and yet it is said, that they had been able to be instructed by English-speaking teachers and to have approbated in 4—5 months all the dental science. The examination of such a man in regard to his improvement and capability is without any value and merely a matter of form, that serves only to procure a rich income to the dental colleges, which are private institutions and even without any control and support by the government.

But there is further a number of so-called "American dentists" who have not been in America at all. Many of these pseudo-doctors were already punished in the last years by German Tribunal and obliged to abdicate their title, received "in absentia." They had bought their titles for a few dollars from one of these American bogus-parties which have a good trade with their doctor-diplomas. The most known of them is the Philadelphia University, Livingstone University and Wisconsin Dental College. In defiance of these detestable cheats, always told and judged in German dental journals, yet they have got a great extension in Germany, thus that without help of a law the oppression of this cheat cannot be expected. It is a matter of course that the reputation of the dental profession is dishonored by such cheats. The ministers of culture at Berlin has them also in the last time acknowledged this great mischief in the region of dental practice, by the action of the writer of these lines, and measures for their regulation will follow.

We expressly speak of the great merits that the U. S., of North America, have gained about the development of dental science and acknowledge it willingly. The influence of the American school has been of great importance twenty years ago, not for dental science in Germany alone, but for dental science in general. Just these circumstances may have been the reason, that nowadays the above said mischief is possible, and that those dentists, provided with such bought doctor-diplomas enjoy the public confidence.

The high degree of development which dental science gained in America prematurely, was also the cause that so many German dentists (the writer of these lines too) went over to America, after having finished their accomplishment at home, (and yet still go) to enrich their experiences and intuitions, like young German physicians go to forcign universities to gather further experiences. It shall not be unsaid that among the American dentists who practice in Germany, and are natives and had a regular accomplishment in their fatherland are many clever and educated men. But the most of the people who nowadays are visiting the dental colleges are such people who are not admitted to a studio in Germany because it is not to be expected that they can study with any success.

For all these persons it is only a matter to avoid the fixations current for the obtainment of the dental approbation in Germany. The same may be said of all those who have got their approbation in Belgium, Holland, Swltzerland, etc., and from all the dentists who approbated after 4—5 months being passed, there is no more to expect than from the great number of dentists or teeth-artists which did not approbate at all. How much damage is done to the people, you may readily see at their enlarged manner of administering laughing-gas and chloroform for the performation of painless operations. In our fatherland to dental science was unhappily not devoted the necessary attention in former times. First by the instalment of the fixations, current in 1869, it was higher esteemed. In the latest time dental science has gained a very great signification, not alone because the amount of people suffering from teeth-sickness increases daily, and the most possible conservation of unhealthy teeth that require a scientifical treatment is acknowledged as the principal matter in dentistry, but also because the improved medical knowledge has shown new relations between diseases of teeth and other sufferings, namely, certain nerves and important organs, (eyes, ears, etc.), thus they are looking forward to repair in dental science and devote to the accomplishment of dentists double care, what in former time had been omitted. This struggle has led to the reconcilment of a want felt since a long time. At Berlin and Leipzig two chairs for dentistry are now established which enables the students to obtain a punctual, theoretical, as well as mechanical, accomplishment. It is to expect that still other universities establish such chairs and that particularly the universities in south Germany will follow the example. Will these new instalments fulfil their purpose if we remain in the dark and mixed situations, where it is possible for any one in obtaining a worthless diploma in a foreign country, through which he can avoid the examination and prove his capability, which is necessary for the success in scientifical surgery? We don't believe that. If we look for the reasons why just in dentistry we see these bad consequences from the liberty of trade, so we find that the motive is to be found in the ignorance of the people, even in the highest classes not to be enabled to distinct scientific dentistry from quackery; while on the other hand, distinction between an approbated physician and a quack, they know very well. Of course, there are still some other reasons. The number of dentists was very small in Germany in proportion to German physicians at the instalment of liberty of trade. Even the uncleverest "teeth-artist" could make money particularly as people did not know anything about right treatment of teeth, through this ignorance they could take advantage very easily. The universal well-known fear for operations upon teeth may be the reason that so many people fall into the hands of quacks, persuaded by debauching advertisements. It is a very interesting fact that practice in dentistry even in the "liberally" Great Britain and in the U. S., of North America, is subjected to far more restrictions than our laws allow, although they are not taken as liberal, and yet they allow the greatest liberty in the region of medical science. In none of these countries it is allowed that a medical person, approbated in another state, can practice in dentistry or as a physician.

It is therefore reasonable that for some time not only in dentistry but also in other branches of medical science efforts have been made to go a step backwards in the rules of the laws, through which great advantages would be gained by the public as well as the faculty.

By all means it is necessary to revise the laws of dentistry in Germany, so it does not fall still more into the hands of quacks and stop the further development of the same.

Hof Zahnarst,
DR. ADOLF PETERMAN,
Frankfort a—m.

---

